# Supplementary material for: How Chanting Relates to Cognitive Function, Altered States and Quality of Life
Source: Brain Sci. 2022 Oct 27;12(11):1456. doi: 10.3390/brainsci12111456 (PMC9688188; doi:10.3390/brainsci12111456)

Supplementary Materials

Table S1. Distribution of Countries.

Country	Frequency	Percentage
Australia	209	45.8
Austria	8	1.8
Bahrain	1	0.2
Belgium	2	0.4
Brazil	1	0.2
Bulgaria	1	0.2
Canada	21	4.6
France	3	0.7
Germany	11	2.4
Hong Kong	1	0.2
Hungary	1	0.2
India	15	3.3
Ireland	2	0.4
Israel	1	0.2
Italy	1	0.2
Malaysia	4	0.9
Mexico	1	0.2
Nepal	1	0.2
Netherlands	8	1.8
New Zealand	5	1.1
Pl	1	0.2
Romania	1	0.2
Scotland	1	0.2
Singapore	3	0.7
South Africa	1	0.2
Spain	2	0.4
Sweden	2	0.4
Thailand	1	0.2
The world	1	0.2
Turkey	1	0.2
UK	21	4.6
USA	124	27.2
Total	456	100

Table S2. Distribution of Nationalities

Nationality	Frequency	Percent
Aboriginal and European	1	0.2
American	115	25.2
Argentina	1	0.2
Australian	176	38.6
Austria	6	1.3
Bahraini	1	0.2
Bangladeshi	1	0.2
Belgian	3	0.7
Brazilian	6	1.3
British	22	4.8
Bulgarian	1	0.2
Canadian	21	4.6
Chilean	1	0.2
Colombian	1	0.2
Czech	1	0.2
Dutch	9	2
Fijian	1	0.2
French	5	1.1
German	13	2.9
Greek	1	0.2
Guyanese	1	0.2
Hispanic	1	0.2
Hungarian	1	0.2
Indian	16	3.5
Indo American	1	0.2
Iranian	1	0.2
Irish	3	0.7
Israeli	1	0.2
Italian	2	0.4
Latina	1	0.2
Lebanese	1	0.2
Lithuanian	1	0.2
Malaysian	5	1.1
Mexican	1	0.2
Mixed	1	0.2
Nepalese	2	0.4
New zealander	1	0.2
New Zealander	7	1.5
other	1	0.2
Pakistani	1	0.2
Pl	1	0.2
Polish	4	0.9
Portuguese	1	0.2

Romanian	1	0.2
Russian	1	0.2
Singaporean	3	0.7
Spanish	1	0.2
Swedish	4	0.9
Swiss	1	0.2
Taiwan	1	0.2
Turkish	1	0.2
Ukrainian American	1	0.2
Welsh	1	0.2
Total	456	100

Table S3. Distribution of Religions

**Religion**	**Frequency**	**Percent**
Monotheistic	64	14.04
Dharmic	73	16.01
Buddhist	54	11.84
Multi-Faith	43	9.43
Spiritual	65	14.25
Vedic	36	7.89
Secular	59	12.94
Other	62	13.6
Total	456	100

Table S4 Regression Table.

	Beta estimate	Standard Error	t-stat	p-value	Structure Coefficient	Squared Structure Coefficient	VIF
**(Intercept)**	8.55E-16	0.036418161	2.35E-14	1			
**experience**	-0.02039934	0.037833296	-0.539190148	0.59002531	0.0898	0.0081	1.0769
**regularity**	0.188463016	0.040833525	4.615399178	5.14E-06	0.5086	0.2587	1.2544
**practice duration**	0.018884842	0.038179092	0.494638314	0.62109956	0.1659	0.0275	1.0966
**sound**	0.033006207	0.037776621	0.873720482	0.38274175	0.2419	0.0585	1.0736
**devotion**	0.075681634	0.045264029	1.672003941	0.09522648	0.5063	0.2564	1.5414
**intention**	0.031291017	0.045063745	0.694372326	0.48781109	0.4707	0.2216	1.5278
**mystical experience**	0.357752101	0.043580078	8.209074346	2.43E-15	0.8222	0.6761	1.4288
**flow**	0.164223835	0.043968661	3.735020178	0.00021209	0.6892	0.475	1.4544
**mindfulness**	0.199740076	0.050619668	3.945898578	9.24E-05	0.4798	0.2302	1.9277
**Mind wandering**	0.102775484	0.048448477	2.121335715	0.03444576	-0.2199	0.0483	1.7659

Number Observations	456
error degrees of freedom	455
Root Mean Squared Error	0.778
R-squared	0.409
F-statistic vs. constant model	30.7
p-value	5.54E-45
Adjusted R-squared	0.395
Number Observations	456
error degrees of freedom	455
Root Mean Squared Error	0.778
R-squared	0.409
F-statistic vs. constant model	30.7
p-value	5.54E-45
Adjusted R-squared	0.395

Figure S1. Distribution of Countries Pie Chart

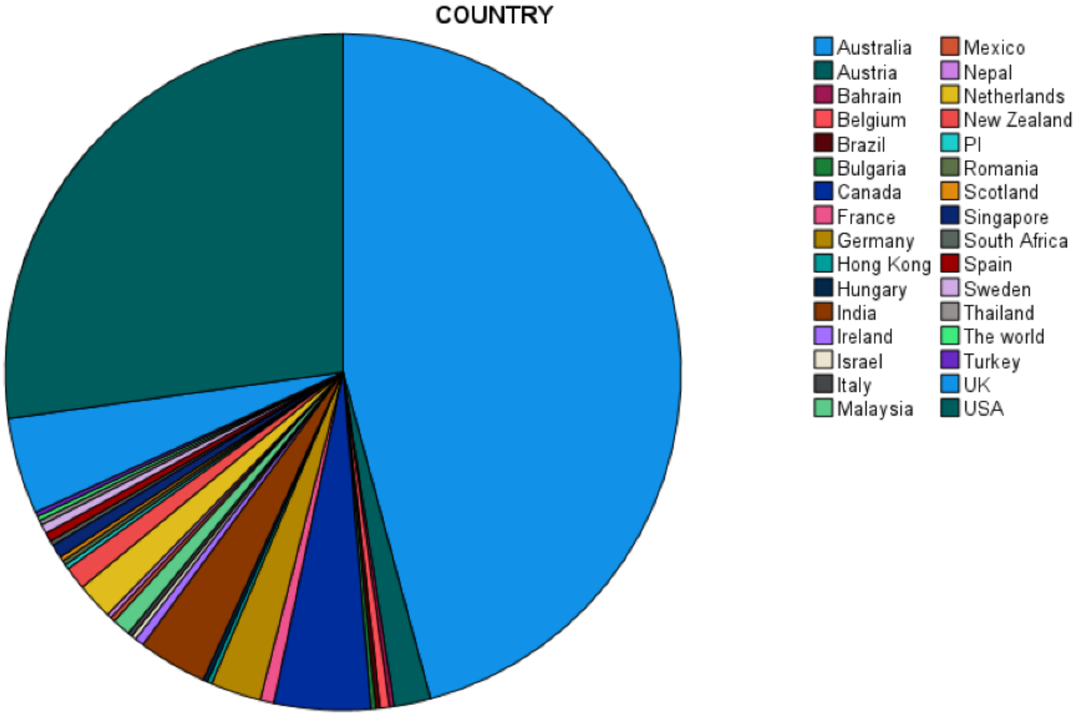
COUNTRY
Australia
Austria
Bahrain
Belgium
Brazil
Bulgaria
Canada
France
Germany
Hong Kong
Hungary
India
Ireland
Israel
Italy
Malaysia
Mexico
Nepal
Netherlands
New Zealand
PI
Romania
Scotland
Singapore
South Africa
Spain
Sweden
Thailand
The world
Turkey
UK
USA

Figure S2. Distributions of Nationalities Pie Chart

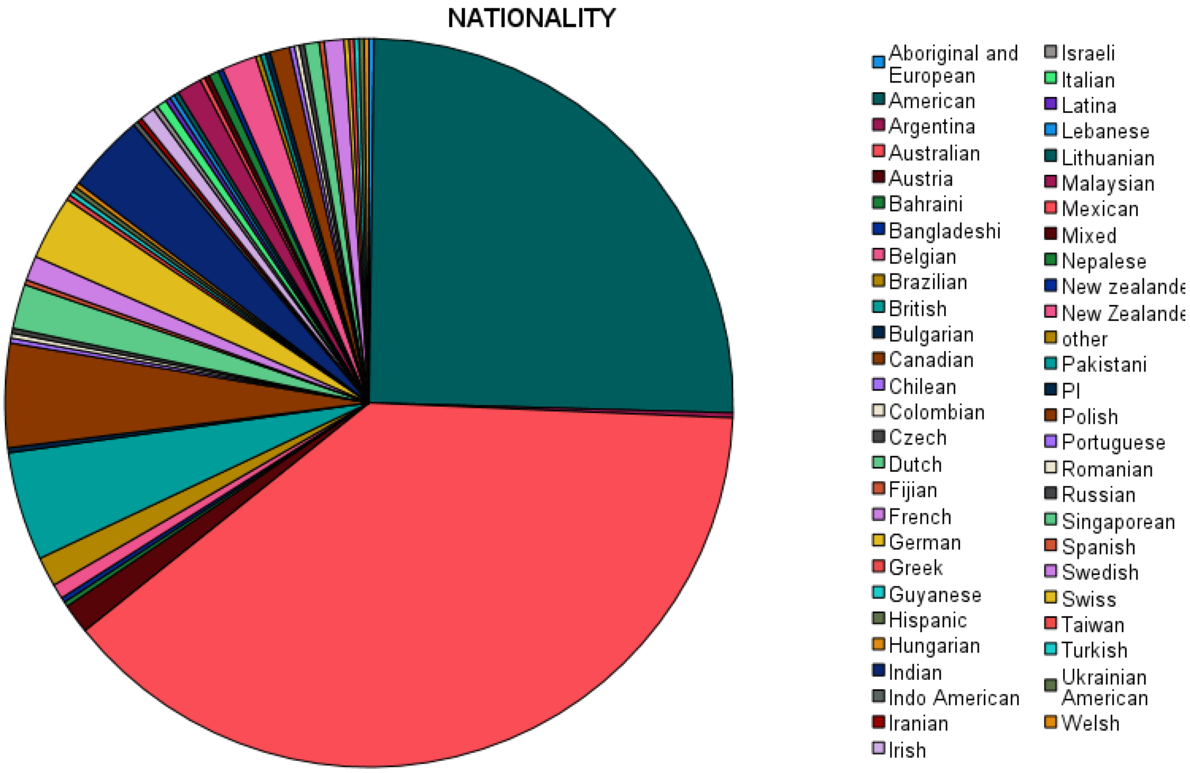

Figure S3. Distribution of Religions Pie Chart

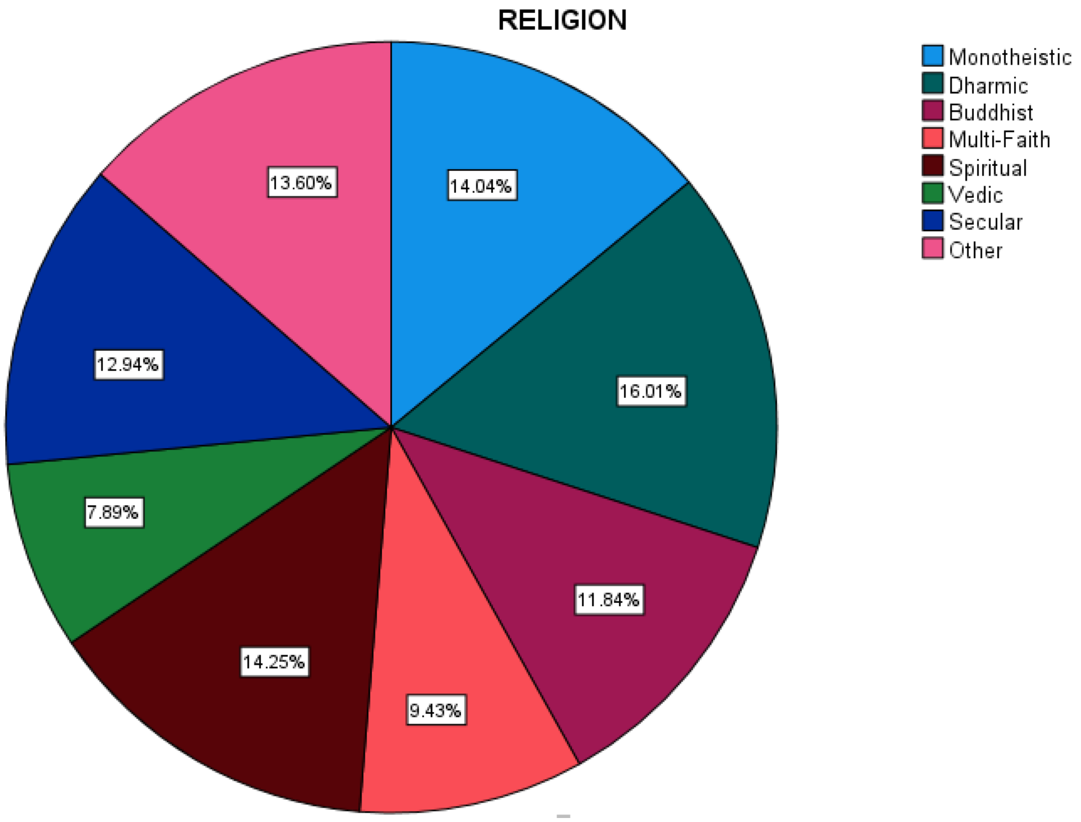

Supplement: Supplementary file 1 [file brainsci-12-01456-s001.zip › brainsci-1972952-supplementary.pdf]
